# Analysis of seroprevalence and risk factors for syphilis and HIV among female sex workers and transgender individuals in different cities of Sindh, Pakistan

**DOI:** 10.1371/journal.pone.0312683

**Published:** 2025-01-24

**Authors:** Sharaf A. Shah, Maria Zubair, Altaf Soomro, Rasheed Sheikh, Alnara Zhamalbekova, Syed Hani Abidi

**Affiliations:** 1 Bridge Consultants Foundation, Karachi, Pakistan; 2 Department of Biomedical Sciences, Nazarbayev University School of Medicine, Astana, Kazakhstan; Jiangsu provincial Center for Disease Control and Prevention, CHINA

## Abstract

**Introduction:**

Co-infections of syphilis and HIV have been found to exacerbate the impact on sexual and reproductive health, especially among key population groups such as Female Sex Workers (FSWs) and Transgender Individuals (TGs). The data on the prevalence and determinants of syphilis and HIV in Pakistan, particularly in Sindh province, is limited. This prospective cross-sectional study aimed to determine the seroprevalence and risk factors for HIV and syphilis infections among FSWs and TGs in different cities of Sindh, Pakistan.

**Methods:**

A total of 1120 participants (531 FSWs and 589 TGs) were recruited from Karachi, Sukkur, Hyderabad, and Larkana. Community-based serological testing for HIV and syphilis was performed using Abbot Bioline HIV/syphilis Duo test kits, and sociodemographic and risk factor data were collected through questionnaires. Chi-square and logic regression were applied to determine variables associated significantly with syphilis in TGs and FSWs.

**Results:**

TGs exhibited higher rates of syphilis (16.29%) and syphilis-HIV (6.79%) as compared to FSWs (syphilis: 15.63%; syphilis-HIV: 0.75%). Inconsistent condom use was more common among FSWs (75.32%), and over half reported STI symptoms. Regression analysis showed that for TGs, having five years (adjusted OR: 0.52, p = 0.04) and graduate-level education (adjusted OR: 0.40, p = 0.04) was associated with a lower risk of syphilis, while an income between 30,000 and 50,000 Pakistani rupees (OR: 1.93, p = 0.028) and more than three years in profession (adjusted OR: 2.20, p = 0.04) was associated with a higher risk. For FSWs, five (OR: 0.34, p = 0.03) and ten (OR: 0.02, p = 0.02) years of education were associated with a lower risk of syphilis, whereas an income between 30,000 and 50,000 PKR (OR: 3.05, p < 0.01) and self-reported HIV-negative status (OR: 3.08, p = 0.01) were associated with a higher risk of syphilis mono-infection.

**Conclusion:**

In conclusion, the results of the study show higher rates of syphilis and HIV co-infection among TG compared to FSW from Sind. The study findings provide valuable insights for national health agencies and policymakers to devise data-driven strategies for preventing and controlling syphilis and HIV infections among FSW and TG populations in Sind.

## Introduction

As per the World Health Organization (WHO), sexually transmitted infections (STIs) are a significant public health concern worldwide [1]. Among the STIs, syphilis and HIV are the two major infections responsible for considerable morbidity and mortality, particularly reproductive mortality, worldwide [2]. Syphilis is caused by the bacterium *Treponema pallidum* and is responsible for approximately 6 million new cases annually among people aged 15 to 49 years [3]. Similarly, HIV remains a significant global health issue, accounting for approximately 1.3 million infections and 0.6 million deaths in 2023 alone [4]. Syphilis and HIV share a common transmission route, and these co-infections have a profound impact on sexual and reproductive health. Syphilis can also increase the risk of HIV transmission by 2-5-fold [2].

Certain key population groups, such as Female sex workers (FSWs) and transgender individuals (TGs), exhibit a significant risk of acquiring sexually transmitted infections (STIs), including HIV and syphilis, due to their engagement in high-risk behaviors such as unprotected sex, having multiple partners, drug use, and limited access to healthcare facilities [5]. Consequently, FSWs and TGs are at an increased risk of complications from both infections, such as rapid HIV progression, neurosyphilis, increased mortality rates, congenital syphilis, etc.

The prevalence of syphilis-HIV co-infection varies across regions. For example, the co-infection rate is 2.71% in South Asia, 0.91% in Southeast Asia, 3.55–21.9% in China, 3.1% in Southern Africa, and 10.5% in Eastern Africa [6–8]. The data on the prevalence and determinants of syphilis and HIV in Pakistan, particularly in Sindh province, is limited. One study found syphilis in 0.9% of antenatal clinic attendees in Karachi [9]. Similarly, few other studies reported the prevalence of syphilis to be 13–16% among high-risk groups [2]. Similarly, the syphilis-HIV co-infection rates are also alarmingly high in certain high-risk groups, with one study reporting 63.2% of HIV-positive TGs to have syphilis [10]. Another study reported 59.5% of HIV-infected individuals with syphilis co-infection, with the highest rates observed among Hijra sex workers (90.9%) [2].

The current study aims to fill the gap by providing data on seroprevalence and risk factors for HIV and syphilis infection (and co-infection) among FSWs and TGs in different cities of Sind. The results of this study are expected to help National Health agencies and policymakers in devising data-driven strategies for prevention and control strategies against syphilis and HIV infection among FSWs and TG populations.

## Materials and methods

### Study design and sample collection sites

This prospective, cross-sectional study, conducted between August 2023 and January 2024, included community-based serological testing for HIV and syphilis on two of the major HIV key populations, namely transgenders (TGs) and female sex workers (FSWs). The participants were recruited through the Drop-in service delivery centers established at Karachi, Sukkur, Hyderabad, and Larkana to deliver services, such as STI diagnosis and treatment, HIV testing, behavioral change, and psychosocial support, pre-and post-test counseling, distribution of condoms, lubricants, and information education and counseling material, pre-exposure prophylaxis (PrEP) medicines, etc. to TGs and FSWs.

### Participant recruitment and counseling

For this study, 1120 participants, including 531 FSWs and 589 TGs (males by birth identifying as females), were recruited. In the case of TGs, our outreach workers initially established contact with *Gurus* (leaders for a transgender community) in four major cities of Pakistan, namely Karachi, Hyderabad, Sukkur, and Larkana, who helped in recruiting TGs at the Drop-in centers. In some cases, the sampling was carried out at the houses of the *Gurus*. Similarly, FSWs were recruited through the sex spot owners (*Aunties*). *Aunties* are linked to the pimps or escorts who provide girls for commercial sex work. FSW samples were also collected from Karachi, Hyderabad, Sukkur, and Larkana through our Drop-in centers, while in some cases, the sampling was carried out at the sex spots. The individuals who did not fall into the FSW or TGs category were excluded from the study. The study was approved by the Ethical Review Committee of Bridge Consultants Foundation (BCF/ERC-003/2023).

Participant samples and data were collected only after written informed consent was obtained from each participant. Only those participants who willingly gave their consent were included in the study. After obtaining written consent, our community counselors provided pre-test counseling, comprising information regarding HIV and syphilis, their causes, mode of transmission, high-risk behaviors, the importance of condom use and lubricants, preventive measures, methods of prevention, and their impact on the participant’s quality of life.

### Participants’ data and sample collection

A questionnaire was also administered to collect sociodemographic and risk factor data such as age, educational status, marital status, monthly income, and risk factors such as self-reported HIV status, number of years in the profession, type of sex, condom use per sex, number of clients in a month, history of STI, history of drug use, and blood transfusion. Subsequently, 0.2 mL of whole blood was collected, through finger prick, from each participant and tested for HIV and syphilis using Abbot Bioline HIV/syphilis Duo test kits for the qualitative detection of antibodies to all isotypes (IgG, IgM, and IgA) specific to HIV-1/2 and/or *Treponema pallidum*.

The test results and the participant’s data were anonymized and maintained with complete confidentiality. The results were disclosed only to the participating subjects in the post-test counseling session. The syphilis-positive participants were adequately treated with tab doxycycline 100mg 1 BD for 30 days. The HIV-positive participants were referred to HIV treatment centers and were also counseled for compliance with antiretroviral drugs (ARVs). The participants with negative results were reassured and counseled for changes in high-risk behaviors.

### Statistical analyses

The data was initially entered and cleaned in MS Excel. The cleaned data was summarized using descriptive statistics. Pearson’s χ2 test was used to evaluate differences between the proportions of different variables in TGs and FSWs. Similarly, logistic regression analysis was used to assess the effect of different variables/factors (independent variables) on outcomes (syphilis and HIV seropositivity). A stepwise selection procedure was adopted for regression analysis to determine variables to be included in the final model. Initially, a univariate analysis was performed for each predictor variable to assess its association with the outcome. Variables with a p-value <0.2 in the univariate analysis were included in the multivariate logistic regression model. The likelihood ratio tests were used to evaluate model fit and compare nested models, and p-values <0.05 were considered statistically significant. The odds ratio (with 95% confidence interval (lower-upper) was used to measure association in logistic regression analysis. The statistical analyses were performed using R studio (packages dplyr, tidyr, and stats) and JASP 0.18.3.0. In all analyses, p<0.05 was considered statistically significant.

## Results

### Sociodemographic and risk factor data for FSW and TGs participating in the study

A total of 1120 participants, comprising 531 FSWs and 589 TGs, willingly participated in the research. Most TG (61.96%) and FSWs (67.04%) were 20 to 35 years old, and most did not receive formal education (43.29% and 61.20%, Table 1). FSWs participating in the study were mostly married (77.58%), while most TGs (77.58%) were unmarried. Approximately 63% of TGs and 45% of FSWs were in this sex work profession for over three years (Table 1). Anal sex practice was most common among TGs (40.06%), while most FSWs (82.48%) were practicing vaginal sex. Inconsistent use of condoms was higher among the FSWs (75.32%). More than half of the FSWs (52.91%) reported a history of experiencing signs and symptoms consistent with STIs. Approximately 12% of FSWs and 6.45% of TGs showed a previous history of blood transfusion (Table 1).

**Table 1:** Sociodemographic and risk factor data for the TGs and FSWs in the study. The table shows the number of participants and percentage of TGs and FSWs in different sociodemographic and risk-factor categories. The last column shows p-values, while statistical significance is indicated by *.

Variables	TG (n = 589)	FSW (n = 531)	P-values
	N (%)	N (%)	
**Age**			
Less than 18 years	7 (1.18)	15 (2.82)	p = 0.0002*
18 to 20 years	57 (9.67)	95 (17.89)	
More than 20 years less than 35 years	365 (61.96)	356 (67.04)	
More than 35 years	160 (27.16)	65 (12.24)	
**Location**			
Karachi	292 (49.57)	231 (43.50)	p>0.05
Hyderabad	97 (16.46)	100 (18.83)	
Larkana	100 (16.97)	99 (18.64)	
Sukkur	100 (16.97)	101 (19.02)	
**Education**			
No formal education	255 (43.29)	325 (61.20)	p = 0.0001*
five years education	148 (25.12)	146 (27.49)	
Ten years education	121 (20.54)	51 (9.60)	
Graduate	65 (11.03)	9 (1.69)	
**Marital Status**			
Never married	457 (77.58)	63 (11.86)	p = 0.0024*
Married	130 (22.07)	412 (77.58)	
Divorced	2 (0.33)	28 (5.27)	
Separated	0	8 (1.50)	
Widow	0	20 (3.76)	
**Income**			
Less than Rs. 30,000 per month	252 (42.78)	320 (60.26)	p = 0.0001*
More than Rs. 30,000 less than Rs. 50,000	119 (20.20)	169 (31.82)	
More than Rs.50,000 less than 80,000	119 (20.20)	37 (6.96)	
More than Rs. 80,000	99 (16.80)	5 (0.94)	
**Number of years Since in Profession**			
Less than a year	112 (19.01)	63 (11.86)	p = 0.0042*
More than a year	32 (5.43)	93 (17.51)	
Two to three years	70 (11.88)	131 (24.67)	
More than 3 years	375 (63.66)	244 (45.95)	
**Self-reported HIV status**			
Status unknown	111 (18.84)	95 (17.89)	p = 0.0354*
HIV negative	405 (68.76)	416 (78.34)	
HIV positive	50 (8.48)	8 (1.50)	
Don’t remember	23 (3.90)	12 (2.25)	
**Type of sex**			
Vaginal	40 (6.79)	438 (82.48)	p = 0.0001*
Anal	236 (40.06)	0	
Oral	6 (1.01)	2 (0.37)	
Vaginal,oral and anal	307 (52.12)	91 (17.13)	
**Condom use**			
Always use	158 (26.82)	57 (10.73)	p = 0.01*
Never use	111 (18.84)	45 (8.47)	
Sometimes use	261 (44.31)	400 (75.32)	
Often use	59 (10.01)	29 (5.46)	
**No of clients per month**			
Less than five	190 (32.25)	29 (5.46)	p = 0.01*
5 to 10	113 (19.18)	175 (32.95)	
11 to 20	121 (20.54)	168 (31.63)	
more than 20	165 (28.01)	159 (29.94)	
**Experienced symptoms and signs of sexually transmitted infections such as vaginal discharge, genital ulcer, genital growths, or warts**			
Yes	172(29.20)	281 (52.91)	p = 0.01*
No	365 (61.96)	228 (42.93)	
Don’t Know	52 (8.82)	22 (4.14)	
**Currently, any symptoms and signs of STI, such as vaginal discharge, genital ulcer, genital ulcer, genital growths or warts**			
Yes	90 (15.61)	227 (42.74)	p = 0.0041*
No	467 (79.28)	299 (56.30)	
Don’t Know	32 (5.43)	5 (0.94)	
**Drug use**			
Yes	94 (15.95)	72 (13.55)	p = 0.0001*
No	492 (83.53)	458 (86.25)	
Don’t Know	3 (0.50)	1 (0.18)	
**Blood Transfusion**			
Yes	38 (6.45)	67 (12.61)	p>0.05
No	544 (92.35)	456 (85.87)	
Don’t Know	7 (1.18)	8 (1.5)	

### Seroprevalence and correlates of syphilis and HIV among FSW and TGs

Out of 589 TGs, 40 (6.79%) tested positive for both HIV and syphilis, 96 (16.29%) for syphilis only, and 19 (3.22%) for HIV only (Table 2). Similarly, out of 531 FSWs, 4 (0.75%) tested positive for both HIV and syphilis, 83 (15.63%) for syphilis only, and 5 (0.94%) for HIV only (Table 2). These results suggest that the frequency of syphilis, HIV mono-infection, and syphilis-HIV co-infection were higher among TGs as compared to FSWs (Table 2).

**Table 2:** HIV and syphilis mono- and co-infections among TGs and FSWs. The table presents the frequency of HIV and Syphilis duo test results for the TGs and FSWs part of the study. The last column shows p-values, while statistical significance is indicated by *.

HIV/Syphilis duo test result	TG (n = 589)	FSW (n = 531)	P-values
HIV and syphilis both positive	40 (6.79)	4 (0.75)	p = 0.0001*
HIV negative syphilis positive	96 (16.29)	83 (15.63)	P>0.05
HIV positive syphilis negative	19 (3.22)	5 (0.94)	p = 0.01*
HIV and syphilis both negative	434 (73.68)	439 (82.67)	p = 0.05
Total	589	531	

For both FSWs and TGs, regression analysis could only be performed for syphilis mono-infection, as data in other groups was too imbalanced, making it unsuitable for regression analysis. Among TGs, five years (adjusted OR: 0.52, 95% CI: 0.29–0.96, p = 0.04) and graduate-level education (adjusted OR: 0.40, 95% CI: 0.17–0.96, p = 0.04) and income of >30,000 (~107 USD) but less than 50,000 (~179 USD) Pakistani rupees (adjusted OR: 1.93, 95% CI: 1.02–3.64, p = 0.04) and more than three years in profession (adjusted OR: 2.20, 95% CI: 1.03–4.71, p = 0.04) were significantly associated with syphilis mono-infection (Table 3). Among FSWs, five (adjusted OR: 0.34, 95% CI: 0.17–0.69, p = 0.03) and of ten (adjusted OR: 0.02, 95% CI: 0.09–0.84, p = 0.02) years education, income of >30,000 (~107 USD) but less than 50,000 (~179) Pakistani rupees (adjusted OR: 3.05, 95% CI: 1.68–5.52, p<0.01) and self-reported HIV negative status (adjusted OR: 3.08, 95% CI: 1.30–7.33, p = 0.01) were significantly associated with syphilis mono-infection (Table 3).

**Table 3:** Factor significantly associated with syphilis mono-infection among TGs and FSWs. The table presents the factors associated with Syphilis mono-infection among TGs and FSWs. The table shows the adjusted odds ratio along with 95% CI (lower-upper) and p-values, while statistically significant variables are shown in bold.

**TGs**				
**Variables**	**Adjusted Odds Ratio**	**p-value**	**95% Confidence interval**	
			**Lower bound**	**Upper bound**
Education (Five years education)	**0.52**	**0.04**	**0.29**	**0.96**
Education (Ten years education)	0.84	0.58	0.46	1.55
Education (Graduate)	**0.40**	**0.04**	**0.17**	**0.96**
Income (more than Rs. 30,000 less than Rs. 50,000)	**1.93**	**0.04**	**1.02**	**3.64**
Income (more than Rs.50,000 less than 80,000)	1.44	0.29	0.73	2.85
Income (more than Rs. 80,000)	0.71	0.41	0.32	1.59
HIV status (HIV negative)	0.83	0.55	0.45	1.52
HIV status	0.89	0.85	0.27	2.94
Years in the profession (More than a year)	1.88	0.28	0.60	5.92
Years in the profession (Two to three years)	2.31	0.07	0.92	5.80
Years in profession (>3 years)	**2.20**	**0.04**	**1.03**	**4.71**
Condom use (Never use)	0.55	0.10	0.27	1.12
Condom use (Sometimes use)	0.63	0.12	0.36	1.13
Condom use (Often use)	0.80	0.61	0.34	1.87
**FSWs**				
**Variables**	**Adjusted Odds Ratio**	**p-value**	**95% Confidence interval**	
			**Lower bound**	**Upper bound**
Education (Five years education)	**0.34**	**0.003**	**0.17**	**0.69**
Education (Ten years education)	**0.28**	**0.02**	**0.09**	**0.84**
Education (Graduate)	0.27	0.28	0.02	2.98
Income (more than Rs. 30,000 less than Rs. 50,000)	**3.05**	**< 0.001**	**1.68**	**5.52**
Income (more than Rs.50,000 less than 80,000)	2.05	0.24	0.61	6.84
HIV status	**3.08**	**0.01**	**1.30**	**7.33**
Marital status (Separated)	2.39	0.39	0.32	17.70
Years in the profession (more than a year)	1.57	0.38	0.57	4.32
Years in the profession (two to three years)	0.55	0.26	0.19	1.56
Types of Sex	1.83	0.12	0.85	3.93
Average number of clients (5 to 10)	3.16	0.10	0.81	12.36
Average number of clients (11 to 20)	0.49	0.33	0.11	2.08

## Discussion

The current study aims to fill the gap by providing data on seroprevalence and risk factors for HIV and syphilis infection (and co-infection) among FSWs and TGs in different cities of Sind.

We found a high prevalence of syphilis in both TGs and FSWs (16.29% and 15.63%, respectively, while the overall prevalence of syphilis-HIV co-infection was significantly lower among FSWs (0.75%) but higher among TGs (6.79%). The prevalence of HIV and syphilis observed in our study was lower than the prevalence reported in Pakistan (and other countries). For example, the integrated biological and behavioral survey (IBBS) conducted in 2011 in 19 different cities in Pakistan reported the prevalence of HIV among transgender sex workers to be 5.2%. In contrast, a survey conducted by Raza (11) in Rawalpindi-Pakistan documented HIV and syphilis prevalence among transgender sex workers to be 23% and 76.9%, respectively. An increasing trend of both HIV and syphilis among FSWs has been seen over the last decade. A study from Indonesia has reported the prevalence of syphilis and HIV as 19.3% and 22% among transgender sex workers [11]. A meta-analysis based on studies from the USA, six Asia-Pacific countries, five Latin America, and three European countries revealed the prevalence of HIV among transgender sex workers to be 19.1% [12]. Another study from China showed the prevalence of syphilis and HIV to be 15.0% and 4.7%, respectively [13]. The differences in prevalence may suggest regional variations in the prevalence of HIV and Syphilis [13]. Additionally, the variation in HIV and syphilis prevalence across studies may also be connected to the differences in study methodologies. In the survey conducted in Rawalpindi, the participants were tested by Immuno Chromatography Technique and confirmed by the ELISA method [14]. In the study from China, HIV was screened using ELISA and confirmed by Western blot, while syphilis was tested using Rapid Plasma Reagin and confirmed by *Treponema pallidum* particle agglutination assay (TPPA) [13]. The Abbot Bioline kit used in this study is very effective for screening; however, it may have lower sensitivity than Western blood and TPPA.

Analysis of demographic/risk factor data showed a significant difference in risk factors such as level of education, socio-economic status, self-reported HIV status, frequency of condom use, number of clients, etc., between TG and FSW. The most significant factors contributing to the high prevalence of HIV and syphilis are unprotected sex, multiple sex partners, socioeconomic factors, etc. [15]. We found that inconsistent condom usage (75.32%) and a high number of partners among FSWs lead to a higher incidence of syphilis among FSWs. A study conducted in the Sino-Vietnam border area of Guangxi, China, also highlighted significant rates of inconsistent condom use among FSWs, which correlated with a high prevalence of sexually transmitted infections (STIs) such as HIV and syphilis [16]. This study highlights the persistent challenges in promoting consistent condom use, particularly among FSWs with low socio-economic status, who often have less access to health services and prevention programs and are at increased risk of STIs.

The regression analysis showed education, income, years in the profession, and self-reported HIV status to be significantly associated with the risk of acquiring syphilis. TGs with a low income (between 30,000 (~107 USD) and 50,000 (~179 USD) Pakistani rupees) exhibited 1.93 times higher odds of acquiring syphilis. Similarly, FSWs within the same income range had 3.05 higher odds of acquiring syphilis, indicating a strong association between moderate-income level and syphilis incidents among both TGS and FSWs. Income level is a significant sociodemographic factor directly affecting access to quality healthcare and resources [17]. This finding is supported by a study in China, which also indicated that FSWs with moderate income levels engaged in riskier sexual practices, increasing STI risks [17, 18]. Moderate income could result from an increased number of clients, which exposes the TGs/FSWs to the risk of STI due to factors such as increased frequency of unprotected sex [18, 19]. At the same time, the income may be insufficient to access healthcare [19, 20]. For example, a global study on HIV infection among men who have sex with men found that higher income was associated with an increased risk of HIV due to greater exposure to high-risk situations, whereas lower income was linked to decreased access to healthcare [20]. These findings suggest that while TGs/FSWs may have enough income from various sexual activities, they might lack the financial resources to invest in safe sex practices and access high-quality health services [21]. TGs over three years in the profession showed 2.20 times higher odds of acquiring syphilis. TGs who are in the profession for longer periods may be at risk of increased cumulative exposure to potential STIs over time; additionally, their sexual networks tend to expand, potentially elevating exposure risk, and they may also experience greater economic pressure to accept riskier clients or practices, particularly if aging impacts their earning potential [22–24].

We also found a significant association between five years and graduate-level education (adjusted OR:0.52 and 0.40, respectively) among TGs and five and ten years of education among FSWs (adjusted OR: 0.34 and 0.02, respectively). Education is an important sociodemographic factor that has been positively associated with decreased risk of STI among different population [20, 25, 26]. A higher level of education generally corresponds to increased health literacy and access to healthcare services [27]. Similarly, a study among FSWs in Ethiopia found that lower levels of education were linked to an increased risk of syphilis [27, 28].

Lastly, self-reported HIV-negative FSWs were associated with higher odds (adjusted OR 2.64) of acquiring syphilis. The link between self-reported HIV-negative status and increased risk of syphilis is challenging to explain. On the one hand, it may highlight the unreliability of self-reported HIV status, especially if individuals have not been tested recently, leading to an underestimation of the risks associated with unsafe sex practices due to undiagnosed infections. This issue was also highlighted in a study conducted in Uganda, which found that self-reported results are not always reliable [29]. On the other hand, it may indicate that individuals who self-report as HIV-negative have a false sense of protection and may engage in unsafe sex practices, such as frequent unprotected sex. This observation is supported by a study from Africa that found that self-perceived HIV status was associated with higher risks of unprotected sex [30].

We anticipate certain limitations of the study. This was a seroprevalence survey based on one test kit (rapid test). Due to the financial and logistic constraints, we could not confirm the serological findings using other methods, such as RPR (Rapid Plasma Reagin) or TPHA (Treponema Pallidum Hemagglutination Assay) for syphilis and combo tests for HIV. In the case of syphilis, TPHA/RPR tests are essential in distinguishing between active, past, or treated infections [31]. This may serve as a potential bias, as without these tests, some individuals classified as ‘syphilis positive’ may not have an active infection but rather a previously treated infection or false positive result. However, it is important to mention that the survey focused on screening TGs and FSWs for initial infection detection (and directing them for further testing and treatment) rather than the staging or clinical management of individual cases [24, 32]. Additionally, data was self-reported by the study participants, and the accuracy of the data could have been ascertained using other means. Finally, since the data was highly imbalanced, regression analysis for HIV mono- and HIV-syphilis co-infection could not be reliably performed, creating a gap in understanding about risk factors associated independently with HIV mono- and HIV-syphilis co-infection. However, since HIV and syphilis share the same transmission route and risk factor [20, 33], the variables found to be significantly associated with syphilis may be extrapolated to understand factors associated with HIV mono- and HIV-syphilis co-infection.

In conclusion, the results of the study show high rates of syphilis and HIV co-infection among TG compared to FSW from Sind. This warrants the need for targeted screening programs, focusing on regular testing for both diseases and preventive measures, such as creating means for a) a safe increase in income/sustenance, b) improved health literacy, c) access to health care and affordable STI treatment, and d) accessing safe sex practices, etc. Additionally, given the high rates of inconsistent condom use among FSWs, interventional programs should emphasize the consistent use of protection, providing free or low-cost condoms and lubricants for this group. Similarly, results from this study may help public health organizations to develop more tailored health protection and promotion programs addressing the unique needs of high-risk groups.

## Supporting information

S1 File Raw data from FSW and TGs used for regression analysis. (CSV)
